# Ascorbic acid encapsulated into negatively charged liposomes exhibits increased skin permeation, retention and enhances collagen synthesis by fibroblasts

**DOI:** 10.1038/s41598-018-36682-9

**Published:** 2019-01-24

**Authors:** Lorena Maione-Silva, Elisandra Gava de Castro, Thais Leite Nascimento, Emílio Ramos Cintra, Larissa Cleres Moreira, Bertilha Alves Santana Cintra, Marize Campos Valadares, Eliana Martins Lima

**Affiliations:** 1 Universidade Estadual de Goiás, Itumbiara, Goiás Brazil; 2 Laboratório de Nanotecnologia Farmacêutica e Sistemas de Liberação de Fármacos, FarmaTec, Faculdade de Farmácia, Universidade Federal de Goiás – UFG, Goiânia, Goiás Brazil; 3 Laboratório de Ensino e Pesquisa em Toxicologia in vitro, Tox-In, FarmaTec, Faculdade de Farmácia, Universidade Federal de Goiás - UFG, Goiânia, Goiás Brazil

## Abstract

Ascorbic acid (AA) is widely used in cosmetic formulations due to its antioxidant property and ability to increase collagen synthesis. Here, we encapsulated AA in vesicles with different lipid compositions. Negative liposome charge favored AA skin retention, with accumulation of 37 ± 12 and 74 ± 23 μg/cm^2^ in the epidermis and dermis, respectively, after 6 hours. Drug flux was influenced by the formulation composition, and both the presence of cholesterol and the liposomes surface charge were able to increase the amount of AA crossing the skin. The formulation was stable for at least 30 days and promoted a 7-fold increase in flux compared to free AA. Additionally, liposomes were able to interact better with keratinocytes and fibroblasts membranes. *In vitro* efficacy studies demonstrated that associating AA to these liposomes resulted in increased effectiveness of type I collagen synthesis by fibroblasts and regeneration of UVA-induced damage in keratinocytes. Our results demonstrate the applicability of AA-negatively charged liposomes in promoting AA cutaneous permeation and increasing the retention and flux of this molecule in the skin. This formulation also increased AA stability and effectiveness, opening new perspectives for its application in view of reducing certain skin ageing outcomes.

## Introduction

Ascorbic acid (AA) is a water-soluble molecule with a fundamental role in the maintenance of various biological activities. Among its various functions, the beneficial effects on the skin stand out. AA acts as a cofactor for essential enzymes in the biosynthesis of collagen, especially types I and type III. These molecules are abundantly present in the dermis extracellular matrix and help support the skin. AA is able to increase mRNA levels by directly stimulating the synthesis of collagen in the skin. In addition, it increases the production of metalloproteinases inhibitors, avoiding the degradation of existing collagen^1–4^. AA is also a potent antioxidant, being able to donate electrons and neutralize the free radicals present in the intra and extracellular matrix, avoiding lipid membrane, DNA and proteins damage that would be caused by oxidative stress^5–7^. Furthermore, topical application of AA can provide anti-inflammatory^8,9^ and depigmenting effects^10,11^.

Skin aging occurs when degeneration overlap with regeneration events, reducing skin structural integrity and causing the loss of biological functions^12^. AA effects on the skin layers motivate its use in cosmetic preparations, especially in formulations that inhibit or minimize the effect of skin aging^13,14^. However, cutaneous application of AA is limited by its hydrophilic property and stratum corneum characteristics^15^. This skin layer is the main responsible for the barrier function and consists of corneocytes immersed in a highly organized lipid matrix. Since water corresponds to only 15–30% of the stratum corneum, a hydrophilic substance can hardly penetrate this hydrophobic layer, and consequently permeate to the other layers of the skin^16,17^. Besides, AA is unstable in aqueous media and upon certain conditions such as light exposure, high pH and temperature increase. It is also easily degraded in the presence of certain enzymes and metal ions, generating products with no biological activity^18–20^.

Nanocarriers offer advantages for the topical and transdermal application of molecules. Nanostructured drug delivery systems can, for instance, increase the solubility and diffusion coefficient of molecules in the stratum corneum and also increase molecule stability^21–26^. Liposomes, which are vesicles composed of phospholipid bilayers delimiting an aqueous compartment that can encapsulate hydrophilic substances, are among the most used types of nanocarriers^27,28^. The challenge for encapsulating hydrophilic molecules in liposomes is their tendency to remain outside the vesicles in the aqueous medium in which the liposomes are dispersed, causing low encapsulation efficiency^29^. Liposome production through the dehydration-rehydration vesicle method (DRV) enables the preparation of liposomes with greater encapsulation efficiency^30,31^. In addition to the active compound characteristics, liposome formulation can also directly influence skin permeation. Lamellarity, surface charge, presence of cholesterol, lipid molecular weight and concentration have been related to changes in the retention and flux of molecules through the skin^24,32–36^. In this work, we describe the technological development of a liposomal formulation containing AA. We demonstrate its suitability in increasing AA stability, promoting high interaction with biological membranes, and increasing skin retention and the effectiveness of AA in the treatment of events related to UV-damage related skin aging.

## Materials and Methods

### Materials

Cholesterol (Chol), soybean phosphatidylcholine (PC), 1,2-dioleoyl-3-trimethylammoniopropane (DOTAP) and 1,2-distearoyl-sn-glycero-3-phospho- (1′-rac-glycerol) (DSPG) were purchased from Avanti Polar Lipids (Alabaster, USA). AA (>99%), sucrose, sodium succinate buffer, sodium thiosulfate and tetrabutylammonium hydrogen sulfate were obtained from Sigma Aldrich (St. Louis, USA). Phosphoric acid (85%, v/v) and methanol were purchased from J.T. Baker (Phillipsburg, USA). Vivaspin ultrafiltration systems (Mw cutoff 100 kDa) were acquired from Sartorius Ltd. (Stonehouse, UK). All other reagents used were of analytical grade.

### Preparation of liposomes

Liposomes containing AA (DRV) were prepared using a methodology adapted from Kirby and Gregoriadis^37^. Different molar ratios of PC, Chol, DOTAP and DSPG were used to obtain liposomes with 25 mM PC (DRV1), 200 mM PC (DRV2), 200 mM PC and 50 mM Chol (DRV3), 200 mM PC, 50 mM Chol and 25 mM DOTAP (DRV4) and 200 mM PC, 50 mM Chol and 25 mM DSPG (DRV5) (Table 1).

**Table 1:** Liposomes composition, mean diameter, polydispersity index (PdI), zeta potential, encapsulation efficiency (EE%) and particle count.

	Lipid composition (mM)				Mean diameter (nm) ± SD	PdI ± SD	Zeta potential (mV) ± SD	EE% ± SD	Particle count (particles/mL × 10^13^) ± SD
	PC	Chol	DOTAP	DSPG					
DRV1	25	—	—	—	161 ± 1	0.14 ± 0.01	+1.4 ± 0.2	17.8 ± 0.6	9.0 ± 0.3
DRV2	200	—	—	—	169 ± 1	0.08 ± 0.01	+6.6 ± 0.5	23.2 ± 1.4	24.6 ± 0.7
DRV3	200	50	—	—	171 ± 3	0.12 ± 0.03	+4.8 ± 0.2	31.2 ± 3.9	57.9 ± 2.0
DRV4	200	50	25	—	190 ± 3	0.17 ± 0.02	+50.1 ± 0.8	58.1 ± 4.0	223.0 ± 8.0
DRV5	200	50	—	25	173 ± 2	0.11 ± 0.04	−44.0 ± 5.0	57.8 ± 2.9	233.0 ± 9.0

Formulations were prepared using the dehydration-rehydration vesicle method, and measurements were made after freeze-drying with sucrose (1:2.5 PC:sucrose, molar ratio). SD = standard deviation; PC = phosphatidylcholine; Chol = cholesterol.

Predetermined amounts of each lipid were dissolved in chloroform, mixed and evaporated to dryness under reduced pressure in a rotary evaporator. Lipid films were hydrated with 0.1 M succinate buffer adjusted to pH 3.0 with phosphoric acid and containing sucrose at the final molar ratio 1:2.5 (PC:sucrose) for optimal cryoprotection. The dispersions were vortexed and extruded ten times through 200 nm polycarbonate membranes. For AA incorporation, liposomes were mixed (1:1, v/v) with AA solution in 0.1 M succinate buffer adjusted with phosphoric acid to pH 3.0. The liposome and AA mixtures were maintained at −20 °C for 24 hours and then freeze-dryed for 24 hours. The dry powders were rehydrated in three steps. First, 1/10 of the original aqueous medium volume was added. The dispersion was stirred for 2 minutes and incubated for 30 minutes. The previous step was then repeated. Finally, the remainder of the dispersant volume was added and the samples were again stirred and kept still for 30 minutes. Acidified water (pH 3.0) was used for the first two steps, and 0.1 M sodium succinate buffer (pH 3.0) was used for the third step. AA final concentration was 5% (w/v).

### Liposomes mean diameter, zeta potential and colloidal stability

Particles mean hydrodynamic diameter, expressed as intensity, and polydispersity index (PdI) were determined using a Zetasizer Nano ZS (Malvern Instruments, Malvern, UK). Liposomes were diluted in water (1:20, v/v) before each measurement. Zeta potential measurements were performed using Zeta Plus (Brookhaven, Long Island, USA), previously diluting the formulations in 1 mM KCl solution (1:30). pH was measured using a digital pH meter (PG1800, Gehaka). All measurements were carried out in triplicate at 25 °C for at least three independent preparations.

The formulation containing PC, Col and DSPG was selected for stability studies and monitored for 30 days after storage at 4 and 25 °C. Amber containers were used to store the samples, which were filled with the formulations up to 2/3 of the flask volume. Liposomes mean diameter, PdI and pH were evaluated, as well as encapsulation efficiency and AA content. All tests were performed in triplicate.

### AA quantification and encapsulation efficiency

AA quantification was performed using a high performance liquid chromatography and diode array detection (HPLC-DAD) method previously developed and validated by our group. All analyzes were carried out on a 1260 HPLC system equipped with a diode array detector (Agilent Technologies, Santa Clara, USA). Tetrabutylammonium hydrogensulfate solution (10 mM) was used as mobile phase at 1.0 mL/min. Peaks were analyzed at 245 nm after injection of 20 μL samples. Separation was achieved using a C18 250 × 4.5 mm, 5 μm Zorbax XDB column and C18 12.5 × 4.6 mm Zorbax XDB pre-column (Agilent Technologies, Santa Clara, USA), both maintained at 25 °C. A water/methanol 4:1 (v/v) mixture, containing 0.02% (w/v) sodium thiosulphate and acidified with phosphoric acid to pH 3.0, was used as diluent for both in liposome analysis and AA skin extraction. For the bioanalytical application, method selectivity and accuracy/recovery were additionally verified. Accuracy/recovery was tested by adding known amounts of the analyte to skin samples, with determination being made for two concentrations (5 and 50 μg/mL) and in triplicate. As for the skin permeation study, AA was recovered from the samples by vortexing for 1 min and ultrasonication for 20 min. Limits of quantification and detection were calculated as 232 ng/mL and 70 ng/mL, respectively.

AA encapsulation efficiency in liposomes (%EE) was calculated as follows: %EE = (mg encapsulated AA/mg of AA initially added to the formulation) × 100. Non-encapsulated AA was quantified after sample ultrafiltration (100 kDa) and centrifugation at 2500 × *g* for 10 minutes. Filtration parameters were validated with AA recovery >98%. Analyzes were performed in triplicate.

### Particle count using nanoparticle tracking analysis (NTA)

Particle count in liposome dispersions was accomplished by nanoparticle tracking analysis (NTA) (NanoSight NS500, NanoSight, Amesbury, UK). Formulations were diluted 1:1,000,000 with ultrapurified water before analysis and automatically injected into the sample compartment. NTA 3.1 software was used for data capture and analysis. Measurements were performed in triplicate.

### Morphological analysis by TEM

Morphological analysis of liposomes was performed by transmission electron microscopy (TEM). Liposomal dispersions encapsulating AA were diluted 100-fold after hydration with ultrapurified water. Aliquots of 20 μL were deposited on formvar-coated 300 mesh copper grids and dried for 10 minutes. The excess formulation was absorbed with filter paper and the samples were subjected to negative staining with one drop of 2% (w/v) uranyl acetate solution. Samples were again dried for 10 minutes and excess reagent was removed with filter paper before analysis in a Jeol JEM-2100 microscope equipped with EDS (Thermo Scientific, Tokyo, Japan).

### *In vitro* skin permeation

Franz type static flow cells equipped with automated sampler (Microette Plus®, Hanson Research, Chatsworth, USA) were used for skin permeation studies. Cells were maintained at 32 °C and 300 rpm shaking. Aliquots of 0.2 mL liposome formulation (DRV) or free AA solution (AA) were placed on the donor compartment, with a diffusion area of 1.86 cm^2^. The receptor compartment was filled with 6.6 mL of 0.1 M succinate buffer adjusted with phosphoric acid to pH 3.0 and containing 0.02% sodium thiosulfate. Pig ear skin with stratum corneum was dermatomized to 750 µm (TCM 300 dermatometer, Nouvag, Sweden) and placed facing upwards between the compartments. Experiments were performed with up to 24 h of permeation.

To determine the amount of AA retained in the skin layers, epidermis (EP) was separated from dermis (D) after immersion of the skin in water at 52 °C for 30 seconds. The samples were cut into small pieces and AA was extracted with 10 mL extraction solvent (water:methanol 4:1 acidified to pH 3.0 with phosphoric acid and containing 0.02% sodium thiosulfate). Vortexing for 1 min and ultrasound bath for 20 min were used to optimize AA extraction from the skin layers. To determine the amount of AA crossing the skin, samples were collected from the receptor compartment at 4, 8, 12, 18 and 24 h, with medium replacement after each collection. Quantification was performed using HPLC. The studies were carried out in 6 replicates. Accumulated amount of AA that permeated through the skin per cm^2^ (Q) was plotted as a function of time (t) to obtain the flux. The kinetic models were obtained by plotting Q × t (zero order), Q × √t (pseudo first order or Higuchi model) and logQ × t (first order). The kinetic model that best fitted the cutaneous permeation of AA was defined based on the *r* value obtained for each case, being the model in which *r* was closer to 1 assigned for each case.

### Cell culture

HaCaT (adult human skin immortalized keratinocytes) were obtained from the Rio de Janeiro Cell Bank (Rio de Janeiro, Brazil) and Balb/c 3T3-A31 (immortalized mouse embryo fibroblasts) marketed by ATCC (Manassas, VA) were used for the *in vitro* studies. Cells were maintained in DMEM medium (Gibco, Grand Island, NY) supplemented with 10% (v/v) heat-inactivated fetal bovine serum (Gibco, Grand Island, NY), 100 μg/ml streptomycin and 500 IU/mL penicillin in a humidified atmosphere with 5% CO_2_ and 37 °C.

### Liposomes internalization

Fluorescent liposomes containing the lipophilic markers L-α-phosphatidylethanolamine-N (lysamine rhodamine B sulfonyl (rhodamine) (Avanti Lipids, Alabama, USA) or coumarin (Sigma Aldrich, St. Louis, USA) were prepared as described previously with 0.02 rhodamine or 0.03 coumarin (mol% of total structural lipids). To evaluate the influence of liposome composition on cell internalization, 1.5 × 10^4^ HaCaT and Balb/c 3T3-A31 cells were plated in individual 35 mm plates and incubated at 37 °C with 5% CO_2_ for 12 h. After removal of the culture medium, cells were incubated with labeled liposome formulations at 100 µM AA diluted with DMEM with 2% FBS for 3 h. The liposome dispersing medium was used as a control. HaCaT and NIH/3T3 cells were then washed, fixed with 4% paraformaldehyde in 10 mM Tris-HCl, and labeled with 0.2 μg/ml 4, 6-diamidino-2-phenylindole (DAPI) (Thermo Fisher Scientific, Waltham, USA). Fluorescence microscopy was performed using excitation/emission of 350–470 nm for DAPI, 578–603 for rhodamine-labeled liposomes and 490–525 nm for coumarin-labeled liposomes. Random images of three fields were recorded by replica with a 20x objective of a Leica DMI4000B microscope (Wetzlar, Germany). The observed fluorescence inside keratinocytes and fibroblasts was considered as a result of the cellular uptake of labeled liposomes.

### Cellular regeneration

HaCaT cells were plated in 96-well flat microplates at 1 × 10^5^ cells/mL and washed with HBSS (Hank’s Balanced Salt Solution) (Sigma-Aldrich, St. Louis, USA) 24 h after incubation. Then 100 μL of this buffer were added to each well. While one plate was protected from light (UV-), the other was exposed to UVA radiation at 10 J/cm² (UV+) using an irradiation chamber (Caron, Marietta, USA). Cells were then washed with buffer solution and treated with AA in its free (AA) or liposomal (DRV) form at 62.5; 125 and 250 μM and incubated for 3 h. DMEM with 2% FBS was used to dilute AA. Cells were washed again with HBSS buffer to remove treatment residues and fresh DMEM medium supplemented with 10% FBS was added to the plates, which were incubated for 24 h. Cell viability was evaluated by MTT assay. MTT solution (5 mg/mL) was added (10 μL/well) and the plates were further incubated for 3 h. After centrifugation at 800 rpm for 5 min, the medium was removed, DMSO was added and the plates were shaken at 25 rpm for 20 min. Absorbance was measured at 545 nm using a microplate spectrophotometer (Thermo Scientific Multiskan^®^ Spectrum, Boston, USA).

### Qualitative analysis of type I collagen by immunofluorescence technique

Balb/c 3T3-A31 cell line is widely used for qualitative analysis of type I collagen since fibroblasts are responsible for the biosynthesis of various extracellular matrix proteins such as collagens^38^. Indirect immunofluorescence was used to analyze these proteins. Cells were plated at 1.5 × 10^4^ cells/35 mm plate and incubated for 12 hours for adhesion. Culture medium was then removed and fresh medium, containing 100 μM AA either in free form or encapsulated in liposomes, was added to the cells. Cell culture medium was added to the control group. After incubation for 48 h cells were washed and fixed with 4% paraformaldehyde in 10 mM Tris-HCl. 3T3 cells were then washed again, and permeabilization of the cell membrane was performed using 0.5% Triton-X for 5 min. Non-specific binding was blocked using PBS with 1% bovine serum albumin (BSA) and 0.1% Tween20, with 60 min exposure at room temperature and under gentle shaking. The primary antibody (polyclonal, rabbit anti-human collagen I 1: 400, Rockland, Limerick, USA) was diluted in blocking solution and incubated with 3T3 cells for 1 h. After rinsing with PBS, cells were incubated with the secondary antibody (polyclonal, Goat anti-rabbit 1: 300, Alexa Fluor® 488, Thermo Fisher Scientific, Waltham, USA) for 30 min, protected from light, at room temperature and under gentle stirring for homogenization. After rinsing with PBS, nuclear staining was performed using DAPI (0.2 μg/mL) and cells were analyzed by fluorescence microscopy (DMI4000B, Leica, Wetzlar, Germany) with 20x objective lens.

### Statistical analysis

Statistical analysis of data was performed using GraphPad Prism version 5.03 software (San Diego, CA, USA). Variation between groups was measured by one-way Analysis of Variance (ANOVA) followed by Students’s T test or Tuckey’s multiple comparison test.

## Results and Discussion

### Liposomes preparation and characterization

Liposomes mean diameter, PdI, zeta potential, EE% and particle count are shown in Table 1. Particles in all formulations had mean diameters smaller than 200 nm and monomodal size distribution. Low PdI values (<0.18) indicated homogeneity in particle size distribution, which is particularly important and tricky to obtain after freeze-drying. The cryoprotectant and its molar concentration were chosen based on preliminary experiments (data not shown), in which different concentrations of sucrose, mannitol, trehalose and lactose were tested. Sucrose at the molar ratio 1: 2.5 (lipid/sucrose) was found to be the most efficient cryoprotectant, maintaining the initial liposome size distribution after freeze-drying and rehydration. EE% results were analyzed as a function of particle number, and a correlation coefficient r >0.99 was obtained for the curve y = 1.737. 10^14^x + 18.654. Since AA is a hydrophilic drug, it can be localized in the external aqueous phase of the formulation or encapsulated in the liposomes interior. The more liposomes are formed, the greater is the amount of aqueous nuclei available for AA to occupy, providing greater EE%. Although a linear ratio was not found, the increase in the lipid amount led to the increase in the number of liposomes formed (eg, DRV1 × DRV2). Increased EE% of hydrophilic substances associated with the increase of lipid amount has been previously described^39,40^. However, in none of these studies the particle number was determined. To our knowledge, this is the first study to draw a direct ratio between liposome number and EE%.

Additionally to the lipid amount, liposome composition such as the type of structural lipid and the presence or absence of cholesterol can influence DRVs EE%^41^. EE% of hydrophilic drugs is also influenced by the preparation technique. Preliminary studies showed that liposomes with the same composition of DRV5, though produced by the lipid film hydration technique, had AA EE% decreased by 20% (results not shown).

EE% of hydrophilic molecules in liposomes obtained by other methods, mainly by the lipid film hydration with the drug-containing solution, is generally quite low^29^. The increased EE% of DRVs is related to the controlled rehydration process, where hydrophilic molecules are in direct contact with the lipids due to the decreased hydrophobic forces, thereby increasing the amount of substance that can be encapsulated^42^.

Liposome formulations DRV1, DRV2 and DRV3 presented surface charge close to zero due to the neutral electric charge of the PC used in their preparation. The addition of a cationic lipid, DOTAP, to DRV4 increased the surface charge to +50 mV, whereas, as expected, the addition of anionic DSPG provided DRV5 liposomes with a negative charge of −44 mV.

### Quantification of AA in the skin

**Figure 1:**
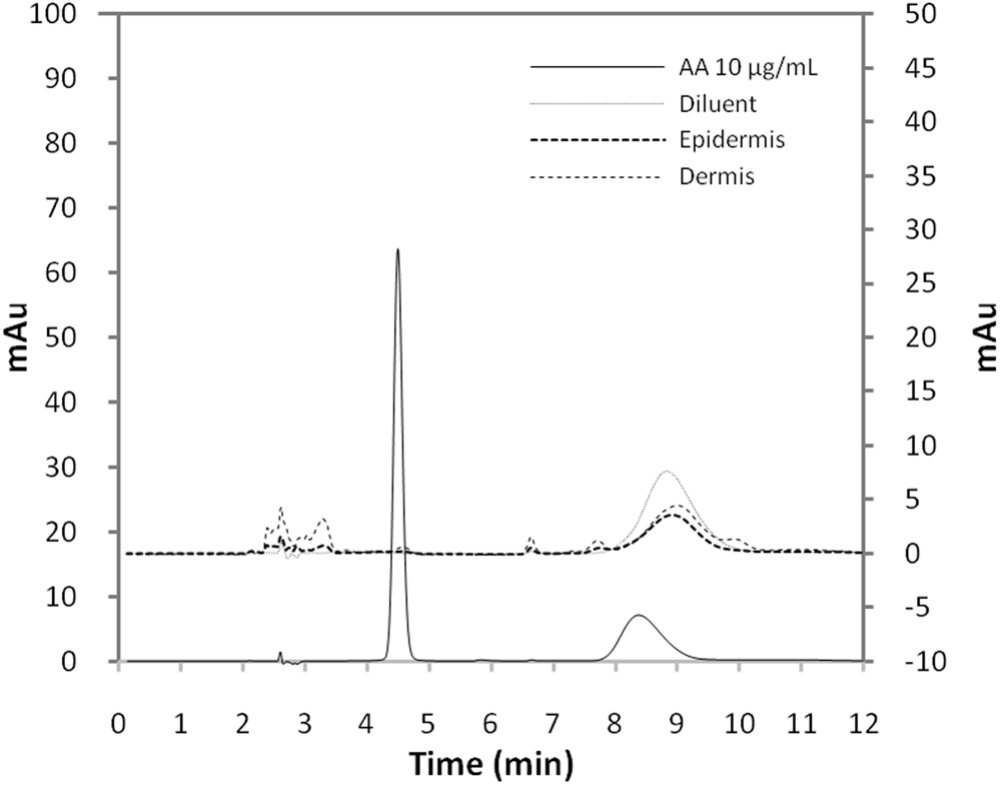
Representative chromatograms obtained using HPLC-DAD for AA (10 μg/mL), diluent and pig ear epidermis and dermis. Chromatographic separation was achieved using C18 250 × 4.5 mm, 5 μm column at 25 °C. Mobile phase was 10 mM tetrabutylammonium hydrogensulfate solution, with 1 mL/min flow. Injection volume was 20 µL. Wavelength 245 nm was used for detection.

A comparison between AA, epidermis and dermis chromatograms showed that the developed method was able to quantify AA without interference from endogenous skin compounds (Fig. 1). The method showed accuracy/recovery between 95.7–96.1% for the epidermis and 91.3–101.6% for the dermis, being suitable for quantification of AA in skin permeation experiments.

### Cutaneous permeation of AA

**Figure 2:**
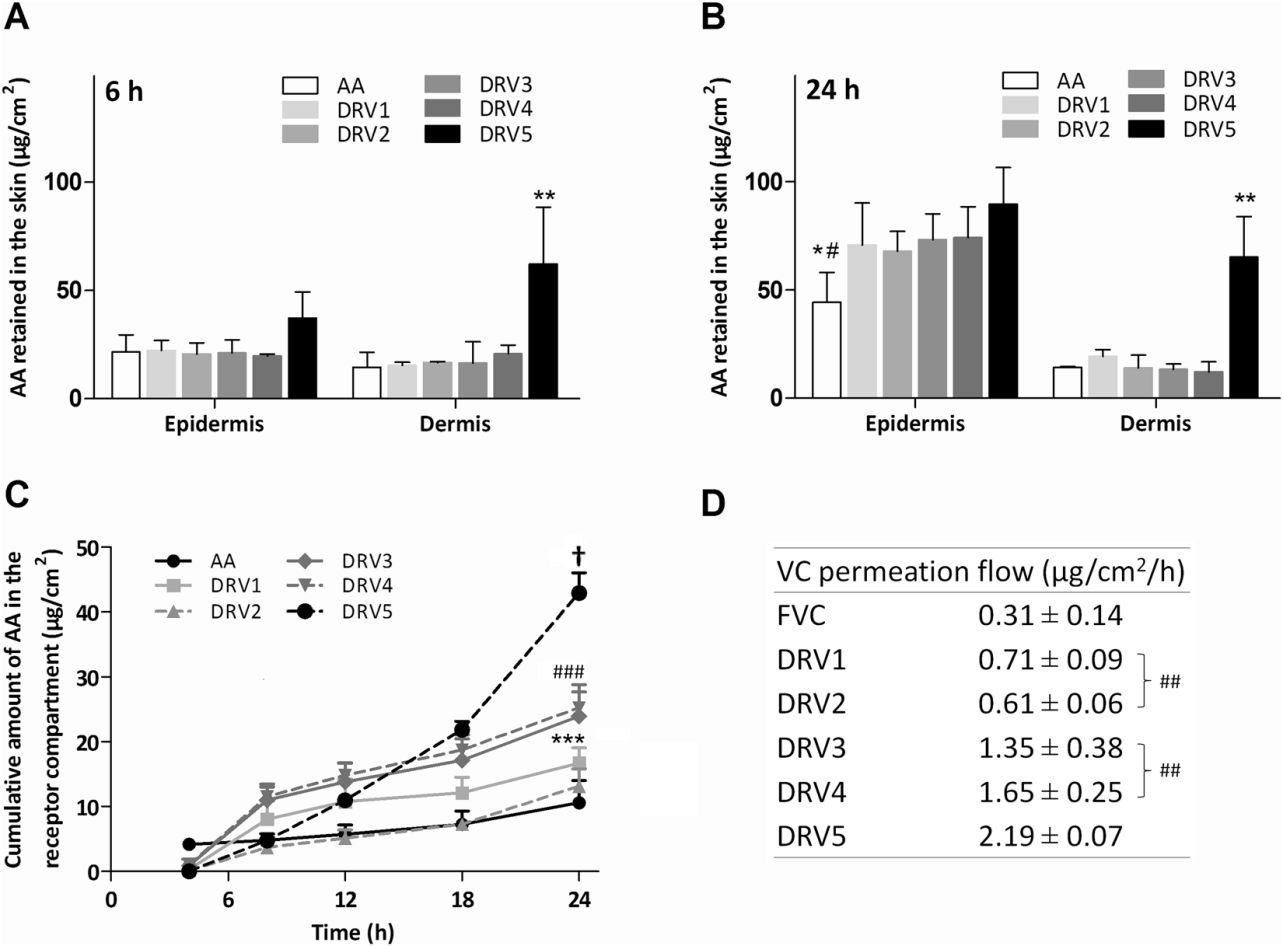
AA permeation and retention results in pig ear skin. AA (**A**) 6 and (**B**) 24 h retention in the epidermis and dermis after application of AA-liposome formulations (DRV) and AA solution (AA). (**C**) Cumulative amount of AA permeated through the skin per cm^2^ during 24 h. (**D**) AA calculated permeation flux. *p < 0.05 free AA *vs* DRV1, DRV2, DRV3 and DRV4 in the epidermis at 24 h. ^#^p < 0.001 AA *vs* DRV5 in the epidermis at 24 h. **p < 0.001 DRV5 *vs* AA, DRV1, DRV2, DRV3 and DRV4 in the dermis at 6 and 24 h. ^†^p > 0.05 DRV5 *vs* DRV1, DRV2, DRV3, DRV4 and AA. ***p > 0.05 DRV4 *vs* DRV2 and AA. ^###^p > 0.05 DRV3 *vs* DRV2 and AA. ^##^p > 0.05 flux DRV 1 *vs* DRV2 and DRV3 *vs* DRV4. Intrinsic AA present in pig skin is not represented in the graph for being under the method’s LOQ. Values are represented as mean ± SD.

Permeation of hydrophilic molecules such as AA through the stratum corneum is related both to their physicochemical properties and to the characteristics of the formulation in which the molecule is dispersed. Here, we evaluated if the amount of lipid (DRV1 × DRV2), presence of cholesterol (DRV2 × DRV3) and liposome surface charge (DRV3 × DRV4 × DRV5) would interfere with the retention of AA in the skin layers in comparison to its free form (AA) (Fig. 2A,B). The results showed that the amount of AA found in the epidermis was not influenced by the formulation composition. However, it increased with time. Considering DRV5, for example, the amount of drug retained in the epidermis was 2.4 times greater at 24 hours (Fig. 2B) than at 6 hours (Fig. 2A). Additionally, AA encapsulation in liposomes favored its retention in the epidermis after 24 hours in comparison to free AA (Fig. 2B) (p < 0.05).

Epidermal retention of AA has a therapeutic interest, since besides the stratum corneum, the epidermis consists of four other layers of living cells that can be favored by AA absorption and metabolization. Furthermore, the epidermis can serve as a reservoir for the drug to reach the next layer: the dermis. There, one of the greatest benefits of AA is expected: the synthesis of collagen by fibroblasts. When free AA was applied to the skin, the amount retained in the dermis was low: 14.5 ± 6.9 μg/cm^2^ after 6 hours (Fig. 2A) and 14.2 ± 0.5 μg/cm^2^ after 24 hours of permeation (Fig. 2B). The same profile was observed after application of DRV1, DRV2, DRV3 and DRV4, indicating that lipid amount, presence of cholesterol and positive surface charge were not able to increase AA retention. However, when AA was encapsulated in negatively charged liposomes (DRV5), a significant increase in the amount retained in the dermis (p < 0.001) was observed. This amount was 5.1 times higher than free AA after 6 hours (Fig. 2A). A similar response was observed after 24 hours of permeation (Fig. 2B). Comparing the permeation of DRV3 and DRV5 formulations, the difference between them being the presence of negative surface charge in DRV5, after 24 hours 5 times more AA was found in the dermis with the use of DRV5 (Fig. 2B).

Few studies have described that negatively charged liposomes may increase drug penetration through the skin. The penetration of betamethasone, a hydrophilic drug, was considerably higher when encapsulated in negative liposomes than in positive and neutral liposomes^22,36,43,44^. Similarly, increased retention may occur upon encapsulation of lipophilic molecules in negative vesicles. For instance, encapsulation of tretinoin in negative liposomes was able to increase its skin retention, besides increasing cutaneous hydration^24^. The improvement in drug penetration with the use of negative liposomes is probably related to the ability of these lipids to promote structural changes in deeper skin layers, favoring the delivery and retention of the drug down to the dermis^36,44,45^.

As mentioned previously, in addition to the lipids characteristics, the properties of the drug/active molecule are also important when skin permeation is evaluated. Typically, hydrophilic molecules have little ability to cross the stratum corneum. However, we observed that even free AA was able to cross this skin layer. This transport was possibly favored by AA small molecular weight (176.12 Da) and its non-ionized form at the pH used for cutaneous permeation studies (pH 3), since AA pKa is 4.2. It has been shown that AA permeation is favored when formulated at high concentrations and at pH < 3.5^46^.

In addition to the amount of AA accumulated in the skin layers, the cumulative amount of AA that permeated through the skin (Fig. 2C) and the influx of AA into the skin (Fig. 2D) were calculated. All liposomal formulations were able to increase AA flux, but in different extents depending on their composition. Although the amount of AA retained in the epidermis and dermis was similar between DRV1 and free AA, for example, it is interesting to note that application of liposomes provided a 129% increase in AA flux through the skin. However, there was no difference in AA flux between DRV1 and DRV2 (p > 0.05), demonstrating that lipid amount was not a factor that interfered with the drug flux (Fig. 2D). On the other hand, the presence of Chol (DRV3) was able to promote a flux of 1.35 μg/cm^2^/h, 4x higher than that of AA (Fig. 2D). Chol presence in liposomes is capable of altering the organization of cutaneous lipids, leading to changes in the penetration and permeation behavior of a molecule in the skin^35^.

There was no significant difference between DRV3 and DRV4 AA flux (p > 0.05), showing that positive surface charge did not improve AA penetration. On the other hand, the presence of negative charges in DRV5 was able to increase AA flux by more than 62%, compared to DRV3 (Fig. 2D). Compared to AA, DRV5 increased by more than 600% the amount of AA crossing the skin barrier (Fig. 2D). This increase is in agreement with previous studies using negatively charges liposomes^22,24,36^. Extrapolating to an *in vivo* application, a formulation capable of inducing greater flux would provide a faster replacement of the AA consumed by skin cells, which would lead to an efficacy increase of the topical product.

It is worth noting that 8 h after DRV5 application the amount of AA quantified in the receptor medium was still small (4.7 ± 0.4 μg/cm^2^) (Fig. 2C). Contrarily, the amount of drug retained in the skin was high, mainly in the dermis (73.9 ± 23.2 μg/cm^2^ at 6 h) (Fig. 2A). Thus, in addition to promoting increased AA flux, DRV5 was also promoted a high retention of this molecule in the skin.

The most suitable kinetic model for free AA was the first-order, where the drug release is directly proportional to the amount of drug remaining (Table 2). In this model, the amount of drug released per unit of time is constantly decreasing, disfavoring AA use as a topical product. Meanwhile, DRV5 presented zero order kinetics, in which the flux is independent of concentration, with a constant drug release per unit of time. Formulations that obey this model are the best pharmaceutical forms for prolonged release, with potential for topical application^47,48^.

**Table 2:** Linear correlation coefficients obtained using first order, Higuchi and zero order kinetic models for AA skin permeation.

Kinetic model	Linear correlation coefficient (r)					
	AA	DRV1	DRV2	DRV3	DRV4	DRV5
Zero order	0.942	0.881	0.959	0.920	0.925	0.954
Higuchi	0.878	0.934	0.935	0.957	0.964	0.894
First order	0.988	0.577	0.684	0.633	0.636	0.918

Diffusion of 0.2 mL aliquots of liposomal formulations (DRV) or free AA solution (AA) through pig ear skin with stratum corneum was quantified over 24 h.

### Effect of liposome composition on cellular uptake

**Figure 3:**
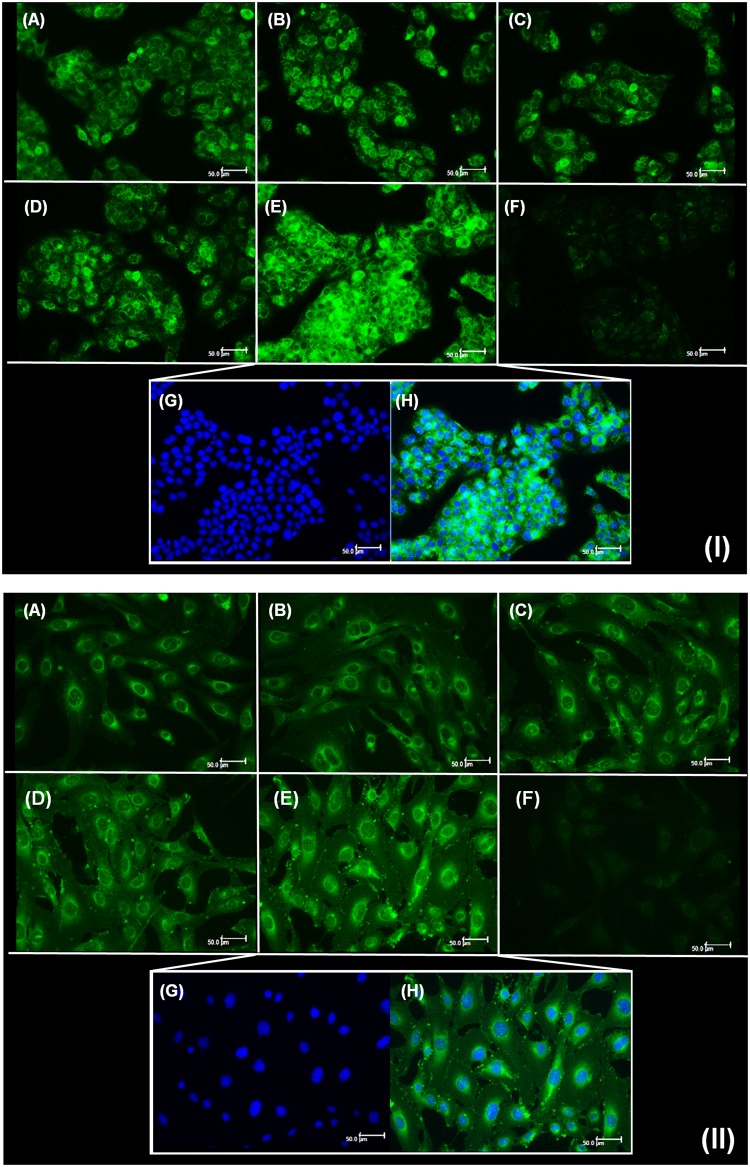
Cellular uptake of coumarin-labeled liposomes by (I) keratinocytes and (II) fibroblasts. Cells were treated for 3 h with (**A**) DRV1, (**B**) DRV2, (**C**) DRV3, (**D**) DRV4, (**E**) DRV5 liposomes or (**F**) AA solution, all containing 100 µM AA. (**G**) Nuclear labeling of DRV5-treated cells and (H) overlap of fluorescence for labeling of nucleus and liposomes. Scale bars = 50 μm.

**Figure 4:**
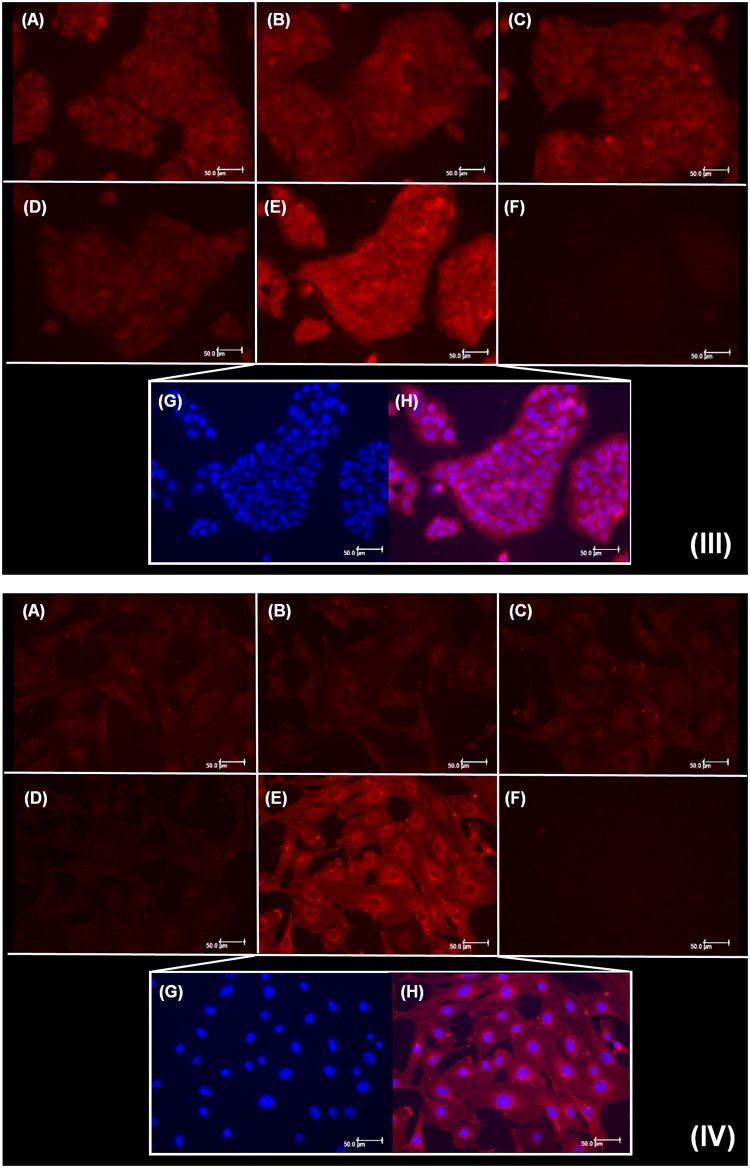
Cellular uptake of rhodamine-labeled liposomes by (III) keratinocytes and (IV) fibroblasts. Cells were treated for 3 h with (**A**) DRV1, (B) DRV2, (**C**) DRV3, (D) DRV4, (**E**) DRV5 liposomes and (**F**) AA solution, all containing 100 µM AA. (**G**) Nuclear labeling of DRV5-treated cells and (**H**) overlap of fluorescence for labeling of nucleus and liposomes. Scale bars = 50 μm.

The evaluation of liposomes cellular internalization can provide explanations regarding the behavior of these particles upon interaction with biological membranes^49,50^, such as in skin permeation studies. In this work, the different liposomes prepared varying lipid content, presence of cholesterol and surface charge, were labeled with rhodamine and coumarin separately, and their uptake by fibroblasts and keratinocytes was analyzed. Fluorescence intensity inside the cells, attributed to the presence of labeled liposomes, was observed in keratinocytes (I) and fibroblasts (II) (Fig. 3). For coumarin labeling, groups treated with DRV5 (3.IE and 3.IIE) presented higher fluorescence intensity, suggesting greater cellular uptake of these liposomes. The difference in DRV5 uptake was even more evident with the use of rhodamine (Fig. 4). It was possible to observe higher fluorescence both in keratinocytes (4.IIIE) and in fibroblasts (4.IVE) treated with DRV5 (Fig. 4).

When DRV4 was labeled with rhodamine (Fig. 4.IIID) a very low fluorescence was observed due to the occurrence of quenching. DOTAP, one of the components of this formulation, has a quaternary amine that possibly influences rhodamine fluorescence, decreasing its intensity^51^. We believe that the low fluorescence observed in 4.IIID and 4.IVD is due to fluorescence quenching and not to low cellular uptake. This observation was confirmed when DRV4 was labeled with coumarin and the quenching adversity was not observed (Fig. 3).

Cell boundaries delimitations, visible in Figs 3 and 4, demonstrate that the liposomes were internalized by both cell lines and occupied the entire cytoplasm.

### PC, Chol and DSPG liposome stability and morphology

DRV5 liposome formulation was selected as the most promising based on the *in vitro* skin permeation and cell internalization studies. The stability of these liposomes after rehydration was then monitored for 30 days at 4 and 25 °C.

Formulation pH remained close to 3 throughout the stability study, being measured at 30 days as 3.0 ± 0.1 and 2.9 ± 0.1 at 4 and 25 °C, respectively. AA content remained close to 100% throughout the analyzed period for samples kept at 4 °C (Table 3). For samples kept at 25 °C, a slight reduction was observed from day 30, nevertheless AA content was >90%.

**Table 3:** Stability of PC, Chol and DSPG (8: 2: 1) liposomes containing AA (5%, w/v), prepared using the dehydration-rehydration vesicle method.

	Storage temp. (°C)	Storage period (days)					
		0	3	7	14	21	30
Mean diameter (nm ± SD)	4	173 ± 2	174 ± 2	174 ± 1	174 ± 1	175 ± 1	176 ± 1
	25	173 ± 2	174 ± 0	175 ± 1	175 ± 1	177 ± 1	178 ± 1
PdI ± SD	4	0.12 ± 0.01	0.11 ± 0.02	0.11 ± 0.02	0.11 ± 0.01	0.11 ± 0.01	0.10 ± 0.01
	25	0.12 ± 0.01	0.12 ± 0.01	0.10 ± 0.01	0.10 ± 0.01	0.10 ± 0.01	0.10 ± 0.01
AA content (% ± SD)	4	100	96.2 ± 5.4	101.1 ± 3.3	98.4 ± 2.0	94.7 ± 3.8	95.0 ± 1.6
	25	100	98.3 ± 7.2	97.3 ± 3.4	97.4 ± 3.5	95.9 ± 1.9	91.3 ± 1.9
EE% ± SD	4	57.5 ± 2.9	49.9 ± 1.7	51.3 ± 3.7	52.3 ± 1.2	49.8 ± 0.5	49.7 ± 4.7
	25	57.5 ± 2.9	49.4 ± 1.3	47.9 ± 1.7	48.9 ± 3.5	41.8 ± 0.6	30.4 ± 3.1

Vesicles mean diameter, PdI, AA content and encapsulation efficiency (EE%) were measured over 30 days upon storage at 4 and 25 °C. SD = standard deviation.

During the first 3 days of the study EE% decreased from 57 to 50%, then remained constant until day 30 when kept at 4 °C. For the samples stored at room temperature EE% underwent a gradual decay during the 30 days, reaching 30%. We attributed this difference to the higher lipid membrane fluidity of liposomes stored at room temperature. Being PC the main phospholipid used as the structural component of the liposomes, its low phase transition temperature (close to zero) favors the liquid-crystalline phase at room temperature and gel phase at 4 °C. The latter presents lower fluidity and therefore a greater capacity to retain the AA in the liposomes aqueous interior compartment^52^. There was no significant change in liposomes mean diameter and PdI (p < 0.001) at both temperatures. It is known that the presence of Chol^35,53,54^ and negative (or positive) charges greater than |30| mV^55,56^ can positively influence liposome stability. As the DRV5 formulation contains Chol and −44 mV surface charge, these factors may have helped maintain liposomes stability in aqueous dispersion for more than 30 days.

**Figure 5:**
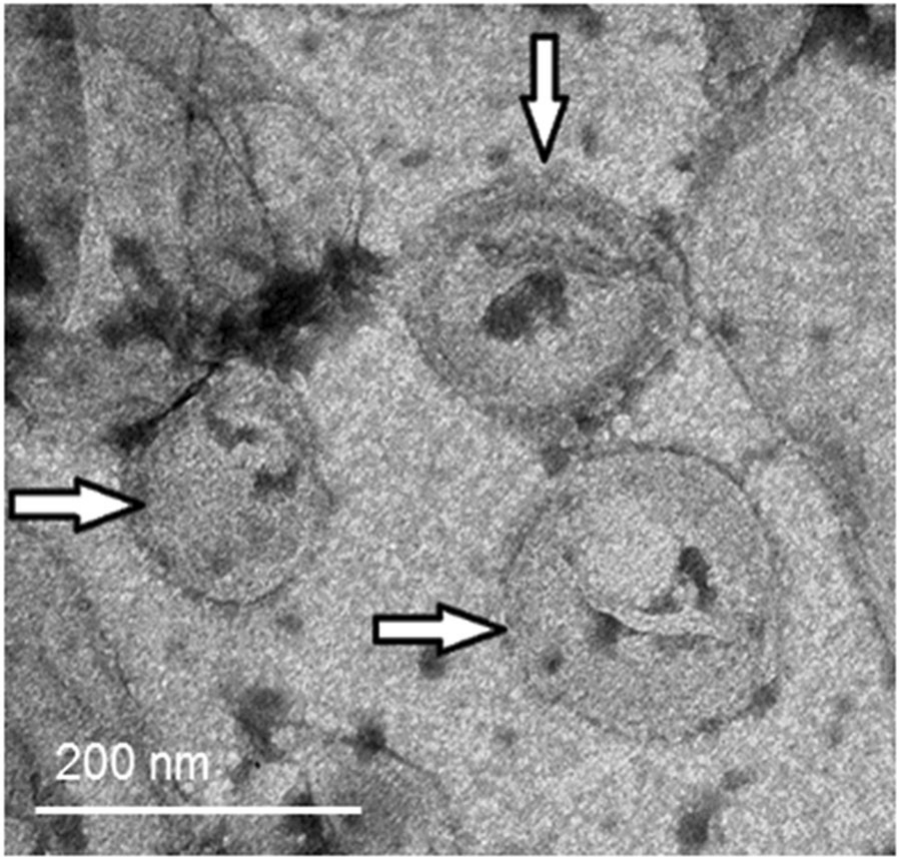
TEM morphological analysis of the DRV5 liposomal dispersion controlled rehydrated after freeze-drying. Arrows indicate the presence of unilamellar vesicles with mean diameters smaller than 200 nm.

Transmission electron microscopy (TEM) was used to visualize the morphology of DRV5 after controlled rehydration (Fig. 5). Uniform and unilamellar vesicles with mean diameters below 200 nm, indicated by the arrows, confirmed the results obtained by DLS.

### Cellular regeneration

**Figure 6:**
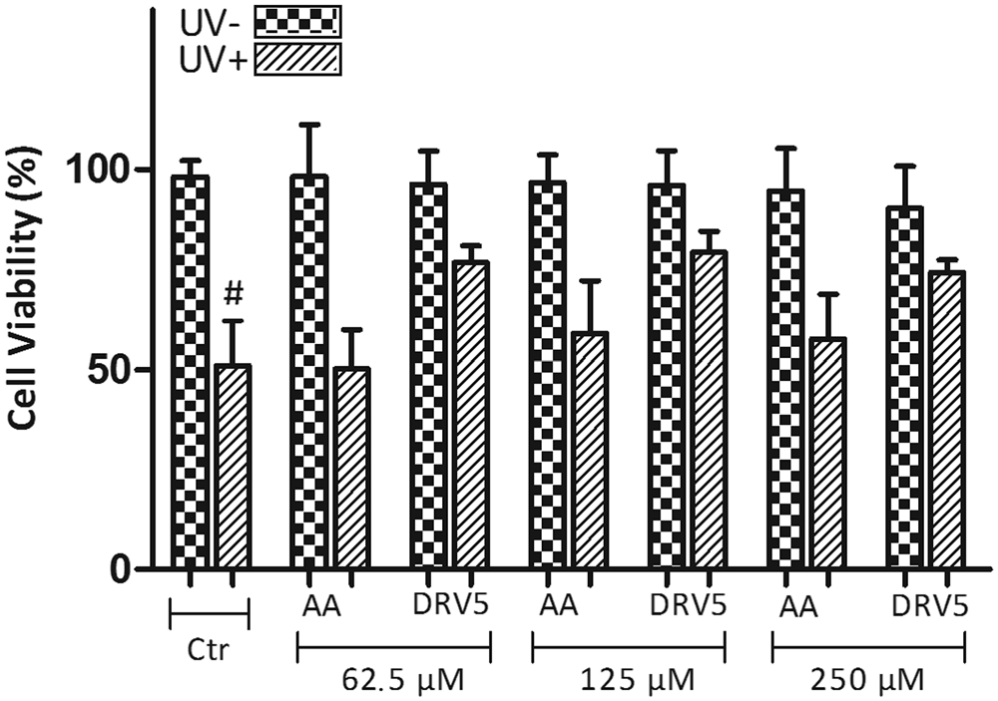
Cell viability of keratinocytes treated with AA at three different concentrations: 62.5, 125 and 250 μM. Cells were incubated with AA either in solution (AA) or encapsulated in liposomes (DRV5), after exposure (UV+) or not (UV−) to UVA radiation. Untreated cells were used as control (Ctr). ^#^p < 0.001 irradiated untreated control (Ctr UV+) *vs* treatment with DRV5 at the 3 concentrations tested (62.5, 125 and 250 μM).

To study cell regeneration, keratinocytes were exposed to 10 J/cm^2^ UVA radiation and then treated with either AA or DRV5. When cells were submitted to UVA radiation (Ctr UV+), a reduction to 51 ± 11% in cell viability was observed (Fig. 6). In the non-irradiated (UV−) and groups treated with AA and DRV5, at all concentrations tested, cell viability remained close to 100% without significant difference (p > 0.05) when compared to the control group (Ctr UV−).

After UVA exposure (UV+) and treatment with AA at 125 and 250 μM a slight and non-significant increase in cell viability was observed compared to the control group (Ctr UV+) (p > 0.05). On the other hand, treatment with AA encapsulated in DRV5 at all 3 concentrations was able to significantly increase cell viability compared to Ctr UV+ (p < 0.001). For instance, after treatment with DRV5 at 125 μM AA, cellular viability was found to be 79.3 ± 5.2%, representing a 55% increase compared to the control (Fig. 6).

Exposure to UVA radiation leads to the formation of reactive oxygen species (ROS), capable of interacting with biomolecules at the cellular level, such as DNA, causing potentially severe changes and consequences for cells^57^. AA is an antioxidant molecule with the ability to prevent and combat the formation of ROS^5^. Under the conditions evaluated by us, AA was only able to recover the damage caused by UVA radiation in keratinocytes when encapsulated in liposomes. The liposomes characteristics enabled greater cell interaction and large drug loading^58^, which could be released more efficiently inside the cells, optimizing AA antioxidant activity. The absence of significant effects on cell regeneration upon AA incubation evidence the advantages of AA encapsulation in liposomes.

### Collagen biosynthesis

**Figure 7:**
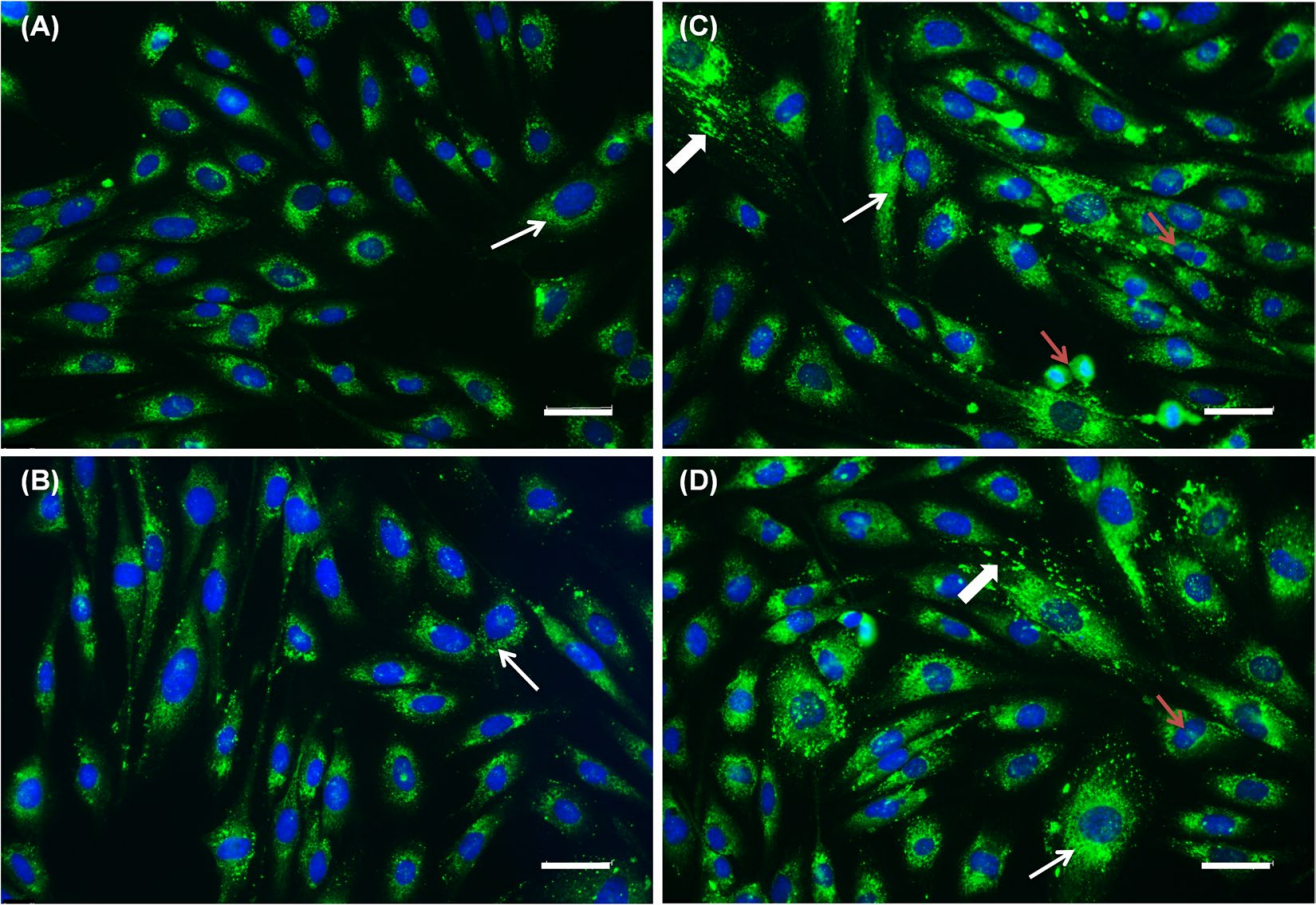
Immunolocalization of type I collagen in fibroblasts treated for 48 h with (**A**) control (cell culture medium), (**B**) 100 μM AA solution, (**C**,**D**) DRV5 containing 100 μM AA. Thin arrows evidence positive intracellular labeling for type I collagen. Thick arrows evidence intense labeling of collagen I in the extracellular matrix. Red arrows show multiple dividing cells in the groups treated with (**B**) AA solution and (**C**,**D**) DRV5. Collagen was labeled with Alexa Fluor® 488 and nucleus with DAPI. Cells were analyzed by fluorescence microscopy with a 20x objective. Scale bars = 50 μm.

The ability of AA to stimulate collagen biosynthesis^1,4,59^ is one of the main expected activities of this drug when used as a cosmetic. Collagen is the main component of the extracellular matrix, with collagen type I being the most abundant (85–90%)^2,60,61^. Among the skin cells, fibroblasts are the ones with the greatest ability to synthesize collagen. In this study, fibroblast were treated with AA in its free form or in DRV5, and the detection of type I collagen synthesis was performed by immunofluorescence. The collagen naturally produced by the fibroblasts (control) can be visualized in Fig. 7A. When treated with AA (Fig. 7B) only a slight increase in the production of collagen was observed, with a more homogeneous distribution throughout the cellular cytoplasm.

A study performed by Heino, *et al*.^62^ demonstrated that treating fibroblasts with 284 μM AA for 48 h stimulated the synthesis of collagen type I, whereas treatment with AA at 100 μM for 72 hours^4^ provided increased transcription of type I, III and IV collagen genes. When fibroblasts were incubated with 100 μM AA, the increase in collagen mRNAs type I and IV expression was only observed after 72 and 120 hours^1^. It is therefore possible that, in the conditions used here for collagen biosynthesis studies, AA concentration (100 μM) and exposure time (48 h) were not enough to allow expressive and complete synthesis of collagen molecules by AA in solution.

In the group treated with DRV5 (7 C and 7D), an increase in the synthesis of type I collagen was observed. It was possible to notice an intense collagen fluorescence in the extracellular matrix, characterized by the excretion of synthesized collagen (Fig. 7C,D, thick arrows). This increase is particularly important *in vivo* as it is the increase of type I collagen in the extracellular matrix that provides the improvement of the skin aspects such as degree of wrinkling, roughness, firmness, smoothness, and dryness^11,13,63^.

In addition, an increased number of dividing cells was observed in the DRV5-treated group, possibly related to the ability of AA to increase fibroblast proliferation (Fig. 7, red arrows)^2^. This increase in fibroblast cell proliferation is also particularly interesting *in vivo*, as in aged skin fibroblasts are compromised, decreasing the amount of collagen being produced^64^.

Negatively charged liposomes improved skin permeation of AA, increasing the amount of molecules being transported to the targeted cells located in different skin layers. The DRV5 formulation could be used for optimizing AA-based treatment and combat of skin aging-related processes.

## Conclusion

Negatively loaded liposomes composed of PC, Chol and DSPG showed stability, high AA encapsulation and *in vitro* affinity for keratinocytes and fibroblasts, as well as improved performance in skin permeation studies. The results demonstrated an increased interaction with the skin, with greater AA flux and retention. Liposomes were able to increase the effectiveness of AA in the production of type 1 collagen and in its antioxidant activity, providing improvement of keratinocytes regeneration *in vitro*. Thus, considering the skin aging damage caused by oxidation and loss of collagen, AA encapsulation in negatively charged liposomes is a promising alternative for high-performance dermatological formulations, and a fresh possibility to rejuvenate and revitalize increasingly demanding skins.

## Data Availability

All data generated or analyzed during this study are included in this published article (and its Supplementary Information files).
